# Diseases potentially related to Flammer syndrome

**DOI:** 10.1007/s13167-017-0116-4

**Published:** 2017-09-13

**Authors:** Katarzyna Konieczka, Carl Erb

**Affiliations:** 1 Department of Ophthalmology, University of Basel, Mittlere Strasse 91, CH-4031 Basel, Switzerland; 2 Eye Clinic Wittenbergplatz, Berlin, Germany

**Keywords:** Primary vascular dysregulation, Retinitis pigmentosa, Leber’s hereditary optic neuropathy, Arterial and vein occlusions, Multiple sclerosis, Tinnitus, Predictive preventive personalized medicine

## Abstract

Flammer syndrome (FS) is a prevalent and mostly benign condition. Subjects with FS seem to have a good life expectancy. Nevertheless, FS subjects are at increased risk for certain diseases, mainly when they are challenged by psychological stress or other stimuli such as coldness. FS is related to ocular diseases, such as normal-tension glaucoma, retinitis pigmentosa, central serous chorioretinopathy, optic nerve compartment syndrome, Leber’s hereditary optic neuropathy, arterial or venous occlusions in the retina, and choroid and optic nerve head, despite the absence of classical vascular risk factors. FS is also related to some non-ocular diseases, such as multiple sclerosis, breast cancer, and altitude sickness. The role of FS in other diseases such as tinnitus, sudden hearing loss, Ménière’s disease, anorexia nervosa, and thyroid dysfunction is currently under investigation. The exact relationship of FS to related diseases however still needs to be established. This may hopefully lead to more targeted diagnostics and personalized treatments.

## Introduction

Flammer syndrome (FS) describes the phenotype of people with a predisposition for an altered reaction of the blood vessels to stimuli like coldness, emotional stress, or hypoxia, together with a group of signs and symptoms, as described previously [1–3]. At least in Europe, the syndrome occurs more often in women than men, in academics more often than in blue-collar workers, and in indoor, more than outdoor workers [4].

The essential component of the FS is primary dysregulation of blood vessels [2]. The term Flammer syndrome [1] was introduced in the scientific literature only recently [1, 5, 6]. Therefore, some aspects described here were labeled in previous publications as vasospasm or primary vascular dysregulation.

## Flammer syndrome in healthy people

Most individuals with FS are healthy, even notably physically and mentally active and usually successful in their professional lives. They normally do not feel sick, because for them, symptoms have more or less always been present, and often their mother or father had the same. Although FS symptoms can be troublesome, FS people normally learn to deal with them, for example, by avoiding coldness, putting on socks at night, increasing salt intake, or adapting doses of drugs.

## Flammer syndrome as a protective factor

Based on clinical experience, FS subjects have a good life expectancy. This can be explained by the low frequency of classical cardiovascular risk factors such as arterial hypertension, hypercholesterolemia, obesity, or diabetes. However, people with increased cold sensitivity in the extremities are additionally protected against metabolic syndrome, probably by an increased level of adiponectin [7]. Nevertheless, FS can be a risk factor for some diseases discussed in this review.

## Flammer syndrome and eyes

A number of ocular signs and even ocular diseases occur more often in subjects with FS [1, 2] than in subjects without FS. While the statistical association is mostly clear, the causal relationship often remains to be established.

### Ocular signs in FS subjects

On average, subjects with FS have reduced or sometimes even missing vascular response to flickering light [8]. Light stimulation of the retina increases blood flow in the smaller retinal vessels (neurovascular coupling) and thereby induces vasodilation in the larger retinal vessels via so-called flow-mediated vasodilation, mediated by the vascular endothelial cells. A reduced response to flickering light, therefore, is an indirect sign of an endotheliopathy. The retinal vessels of FS subjects also exhibit increased spatial vascular irregularity [9], indicating that the dysregulation is not homogeneous along these vessels.

Optic disc hemorrhages, classically observed in patients with glaucoma, particularly normal-tension glaucoma (NTG), occur more frequent in FS patients, sometimes even without glaucoma [10]. J. Flammer first formulated the hypothesis that this is a consequence of a weakened blood-retinal barrier [11]. In subjects with FS, the retinal astrocytes (and most probably the astrocytes in the optic nerve head) are more often or more extensively activated [12]. J. Flammer suggested that activated astrocytes increase the backscatter of light and can therefore be observed clinically in red-free light [12]. Astrocytes also play an important role in neurovascular coupling [13], and therefore their activation may further contribute to the attenuation of the response to flickering light. Astrocytes have their own hemoglobin system [14] for the transport of oxygen from the vessels to the neural axons. When activated, this oxygen transport is reduced, leading to hypoxia in the axons, while the oxygen saturation in the retinal veins increases [15]. Retinal venous pressure is, on average, slightly increased in healthy FS subjects but markedly increased in glaucoma patients with FS [16]. Subjects with FS and particularly glaucoma patients with FS often indicate pain, mostly localized behind the upper lid [17]. This can be explained by a relative hypoxia in the ciliary muscle and the increased pain sensitivity of FS subjects [2]. Rare but very disturbing are scintillations noticed when the eyes are open or closed. FS subjects sometimes describe visual disturbances after sexual activity, lasting for several hours (unpublished data).

### Ocular diseases related to FS

#### Glaucomatous optic neuropathy

FS is one of the risk factors for glaucomatous optic neuropathy (GON) [18–21]. GON is characterized by a loss of neuronal axons and tissue remodeling, resulting in optic nerve head excavation and visual field loss. Primary vascular dysregulation, the essential component of FS, leads to hypoxia, and—even more important—to instable blood flow, which in turn locally increases oxidative stress, an essential component of the pathogenesis of GON. Ocular perfusion in FS subjects is low due to the following facts: low blood pressure, increased vascular resistance, and increased retinal venous pressure; and in addition, is unstable, due to disturbed autoregulation. This pathogenetic concept of J. Flammer has been described in previous reviews [22, 23]. Clinically, such patients with FS can be recognized by symptoms of FS, but often, in the case of glaucoma, by the typical appearance of the optic nerve head, in which the retinal vessels of the optic nerve head are less shifted to the nasal side [5] than in patients with other types of glaucoma.

#### Retinitis pigmentosa

Retinitis pigmentosa (RP) refers to a group of hereditary diseases of the posterior segment of the eye characterized by degeneration of rod and cone photoreceptor cells and the loss of retinal pigment epithelium function. The main symptoms are night blindness and progressive visual field loss, leading to tunnel vision and eventually blindness.

There is a relationship between RP and FS [24–26]. Even though RP has a clear genetic background [27], additional factors can influence the manifestation and progression of the disease [24]. One potential modifying factor is reduced ocular blood flow [28, 29]. Part of this is obviously secondary to tissue atrophy. The fact, however, that blood flow reduction already occurs in the early stages of the disease [30] and is not confined to the eye [28] is an indicator for an additional, primary component. We hypothesized that primary vascular dysregulation or FS, respectively, may be a major cause of this primary component of blood flow reduction [28]. Indeed, recent studies found a relationship between RP and FS [25, 26]. Again, the oxidative stress, induced by the unstable blood flow, might play an important role.

#### Vascular occlusions

##### Retinal arterial occlusions

The classic causes for arterial occlusions are emboli or atherosclerosis and other structural diseases of the blood vessels. Sometimes, however, occlusions occur even in young patients and despite the absence of such risk factors, but in the presence of FS. These occlusions often affect the optic nerve head [31], and less often, the choroid [32], cilioretinal vessel [33], and the retina [2]. Such occlusions are often triggered by major emotional stress. A relationship between Susac syndrome and FS has also been described [34]. Retinal and optic nerve head infarctions have also been described as a perioperative complication in FS patients [35, 36].

##### Retinal vein occlusions

While in the older literature, a retinal vein occlusion was considered to be always a consequence of a thrombosis; J. Flammer hypothesized that it may also be a consequence of a constriction of a retinal vein [37, 38]. There are several causes for such a constriction; the FS is one of them, particularly in younger patients [37]. While a mild constriction just increases the retinal venous pressure, a more pronounced constriction leads to the clinical picture of a retinal vein occlusion [38, 39]. These venous constrictions are induced by vasoconstrictive molecules such as endothelin-1. These molecules can diffuse to the veins from neighboring sick arteries or from circulating blood via fenestrated choriocapillaries into the optic nerve head and adjacent tissue. Endothelin is also produced by local hypoxic tissue. This explains why not just arteriosclerosis, systemic hypertension, and ocular hypertension but also FS increase the risk for vein occlusions.

#### Central serous chorioretinopathy

Central serous chorioretinopathy is a disease characterized by serous retinal detachment and/or retinal pigment epithelial detachment, particularly in the area of the macula, due to leakage of fluid through the retinal pigment epithelium into the subretinal space.

Central serous chorioretinopathy is accompanied by a local vascular dysfunction of the choroid, particularly dilated veins [40]. The cause of this vascular dysfunction is not yet clear. It has been reported to occur more often in Type-A personalities and manifests itself particularly when patients are under stress. Based on our clinical experience, the endothelin-1 plasma level rises markedly in the acute phase and then returns to nearly normal levels within days or weeks. Most, but not all of our patients with central serous chorioretinopathy suffered from FS.

#### Optic nerve compartment syndrome

Optic nerve compartment syndrome [41, 42] is a pathological condition in which cerebrospinal fluid of the subarachnoid space surrounding the optic nerve is partially or totally segregated from the cerebrospinal fluid of the intracranial subarachnoid space, leading, inter alia, to an increase in pressure and thereby also an increase in the diameter of the optic nerve sheath.

The pathogenesis of this condition is not yet clear. In our clinical experience, the majority of patients with the combination of optic nerve compartment syndrome and glaucoma also suffer from FS [2, 42]. FS, via unstable blood flow, increases oxidative stress and thereby may contribute to swelling of the arachnoid trabeculae, septa, and pillars in the subarachnoidal space of the optic nerve. FS may also reduce the outflow of cerebrospinal fluid through the lymphatic vessels. This would explain why treatment of FS with a calcium channel blocker also reduces the extension of the optic nerve sheath diameter in such patients [43].

#### Leber’s hereditary optic neuropathy

Leber’s hereditary optic neuropathy (LHON) is a maternally inherited disorder resulting from point mutations in mitochondrial DNA (mtDNA). The condition can lead to acute or subacute visual loss starting in one eye and involving the other eye after some latency. At the late stage, the optic nerve head is pale with some glaucoma-like excavation. This stimulated discussions whether there might be a potential relationship between LHON and normal-tension glaucoma [44]. Mitochondrial dysfunction is a major component in both diseases, in LOHN due to an mtDNA mutation and in NTG due to oxidative stress induced by unstable oxygen supply. The majority of LHON patients we saw also had FS [2, 45]. We assume that the unstable oxygen supply in FS subjects not only contributes to the development of GON but also increases the risk that the genotype of mtDNA mutations manifests as phenotype of LOHN [45].

## Flammer syndrome and other diseases

### Systemic signs and symptoms of FS

The most prominent, but not mandatory, signs and symptoms of FS are cold hands and/or feet with an increased response to coldness [46] and arterial hypotension [47]. FS subjects often have a low body-mass-index [48]. On average, they need longer to fall asleep and exhibit a phase delay of the circadian rhythm by approximately one hour. They often have a reduced feeling of thirst. This may be due to a slightly increased level of endothelin-1 in circulating blood [49], as endothelin suppresses the thirst center in the brain. FS subjects are generally more sensitive; they have for example increased sensitivity to certain drugs, increased pain sensation, increased smell sensation, and increased sensitivity to high altitude. FS subjects have more frequent headaches and migraines. FS and migraines, however, are two distinct entities that should not be confounded. FS subjects often suffer from tinnitus and have a tendency toward perfectionism. Blood flow cessation in the nailfold capillaries after cold provocation is prolonged. FS subjects have an autonomic imbalance with sympathetic predominance [50]. Nevertheless, the causal relationship with the vascular dysregulation is unclear, as non-autonomic innervated retinal vessels are also involved in FS [2]. FS subjects have altered gene expression [51] and increased systemic oxidative stress [52]. For more details, please refer to previous reviews [1, 2] and other articles in this issue.

### Non-ocular diseases related to FS

#### Multiple sclerosis

Multiple sclerosis (MS) is a chronic, demyelinating, degenerative disease of the central nervous system of unknown etiology. MS patients suffer significantly more often from FS signs and symptoms than do controls [2, 53]. The cause of this relationship remains unknown at the moment. There are two possibilities: MS may induce FS symptoms, or FS subjects may have a higher risk of developing MS. Interestingly, MS patients often indicate they had symptoms of FS before they suffered from MS. J. Flammer et al. hypothesized that FS may sometimes lead to clinically undetected microinfarctions in the central nervous system, which then potentially trigger an autoimmune disease [2]. FS also leads to increased oxidative stress, which in turn could contribute to the pathogenesis of MS. In a later stage of the disease, the inflammations induce a secondary vascular dysregulation which further contributes to the chronic progression of MS [54].

#### Breast cancer and metastatic disease

The relationship between breast cancer and FS is being discussed and investigated [55] and some findings are presented in this special issue. Hypoxia and oxidative stress due to FS may predispose to breast cancer development and progression into aggressive metastatic disease [56–59] 

#### Altitude sickness

Altitude sickness is a general term which encompasses a spectrum of disorders that occur at higher altitudes. The primary cause is the low oxygen level at higher altitude that leads to tissue hypoxia and thereby to an increase of HIF-1 α. This leads to increased expression of several hormones, including erythropoietin and endothelin-1. FS subjects not only have higher plasma endothelin level but also higher sensitivity to endothelin [60]. Symptoms of altitude sickness are more pronounced in FS subjects [2, 61, 62].

### Under investigation

#### Tinnitus, sudden hearing loss

Clinical experience indicates that FS is also often related to tinnitus, sudden hearing loss, and even Ménière’s disease. These potential relationships are presently under investigation.

#### Anorexia nervosa

Clinical experience indicates that FS signs and symptoms often occur in patients with anorexia nervosa. This relationship is currently under investigation. Interestingly, fasting intensifies symptoms in FS subjects, which is in line with the observation that fasting reduces the responses of retinal vessels to flickering light [63].

#### Thyroid dysfunction

Thyroid diseases and dysfunctions are often observed in patients with NTG. Hypothyroidism can be associated with vascular dysfunction such as impaired endothelial- and non-endothelial-mediated vasodilation. We often found antibodies against thyroid gland in euthyroid patients suffering from the combination of NTG with FS. Some glaucoma patients with FS also suffer from Hashimoto thyroiditis.

The causal relationship between thyroid dysfunction and FS is not yet clear. FS may lead to subclinical microinfarction, triggering autoimmunity.

#### Heart diseases

Prinzmetal angina is a vasospastic disease occurring mostly in younger patients. A relationship with FS is likely, but has not yet been studied. Already known however is the fact that FS subjects often suffer from silent myocardial ischemia [2].

#### Whiplash trauma

A whiplash injury is the result of the sudden deceleration or acceleration of the thorax independent of head movement. This type of injury is commonly associated with motor vehicle collisions. As a result of the acceleration/deceleration motion, it is thought that patients may sustain bony or soft tissue injuries. Symptoms caused by the injury are grouped together as “whiplash associated disorders” and encompass neck pain, headaches, dizziness, and sleep disturbances.

We made a clinical observation that patients with whiplash trauma had more and longer-lasting symptoms when they suffered from FS.

## Conclusion

While FS rather protects against certain diseases like metabolic syndrome and its related vascular events, it predisposes for some other diseases, particularly eye diseases.

It remains open at present whether treatment of FS will reduce the risk for such diseases or will slow down its progression. If this is the case, this will lead to predictive diagnostics and preventive treatment tailored to the person.

List of abbreviations: FS, Flammer syndrome; GON, glaucomatous optic neuropathy; LHON, Leber’s hereditary optic neuropathy; MS, multiple sclerosis; NTG, normal-tension glaucoma; RP, retinitis pigmentosa
